# Inferential Communication: Bridging the Gap Between Intentional and Ostensive Communication in Non-human Primates

**DOI:** 10.3389/fpsyg.2021.718251

**Published:** 2022-01-14

**Authors:** Elizabeth Warren, Josep Call

**Affiliations:** School of Psychology and Neuroscience, University of St Andrews, St. Andrews, United Kingdom

**Keywords:** animal communication, primates, social inference, communication cognition, intentionality, inferential communication, cognitive flexibility, social cognition

## Abstract

Communication, when defined as an act intended to affect the psychological state of another individual, demands the use of inference. Either the signaler, the recipient, or both must make leaps of understanding which surpass the semantic information available and draw from pragmatic clues to fully imbue and interpret meaning. While research into human communication and the evolution of language has long been comfortable with mentalistic interpretations of communicative exchanges, including rich attributions of mental state, research into animal communication has balked at theoretical models which describe mentalized cognitive mechanisms. We submit a new theoretical perspective on animal communication: the model of inferential communication. For use when existing proximate models of animal communication are not sufficient to fully explain the complex, flexible, and intentional communication documented in certain species, specifically non-human primates, we present our model as a bridge between shallower, less cognitive descriptions of communicative behavior and the perhaps otherwise inaccessible mentalistic interpretations of communication found in theoretical considerations of human language. Inferential communication is a framework that builds on existing evidence of referentiality, intentionality, and social inference in primates. It allows that they might be capable of applying social inferences to a communicative setting, which could explain some of the cognitive processes that enable the complexity and flexibility of primate communication systems. While historical models of animal communication focus on the means-ends process of behavior and apparent cognitive outcomes, inferential communication invites consideration of the mentalistic processes that must underlie those outcomes. We propose a mentalized approach to questions, investigations, and interpretations of non-human primate communication. We include an overview of both ultimate and proximate models of animal communication, which contextualize the role and utility of our inferential communication model, and provide a detailed breakdown of the possible levels of cognitive complexity which could be investigated using this framework. Finally, we present some possible applications of inferential communication in the field of non-human primate communication and highlight the role it could play in advancing progress toward an increasingly precise understanding of the cognitive capabilities of our closest living relatives.

## Introduction

Communication modifies the behavior of others by altering the psychological state of the recipient. Unlike instrumental actions, which bypass the recipient’s psychological states and act directly on their behavior, communicative acts affect the perception, attention and/or cognition of recipients, and, if successful, subsequently provoke the desired behavior. Consider, for example, an infant chimpanzee who, while clinging to their mother, begins to nurse. The infant is engaged in an instrumental action with a direct effect on the mother’s body, without engagement with the mother’s psychological state. Although the mother could choose to disrupt the infant’s feeding behavior if she did not desire that interaction, the feeding interaction itself is instrumental, not communicative. Conversely, an infant chimpanzee who reaches their hand toward their mother’s back, a ritualized gesture which requests carrying (Hobaiter and Byrne, 2014), is altering the mental state of the mother, who may react to her perception and cognitive processing of this event by lifting the infant onto her back and performing the desired carrying behavior. Although the ultimate outcomes of the two interactions are similar – the infant’s physical needs are met – the proximate mechanisms that permitted these outcomes are fundamentally different. The proximate mechanisms of communication, the alteration of psychological states to influence behavior, are an exceptional lens through which we can probe the levels of cognitive engagement involved in different communication systems.

Psychological states play a central role in all forms of communication, from the wing spots of a butterfly to the courtship display of a gull to linguistic exchanges between humans. These systems of communication differ, however, in their origins and, more importantly for our purposes here, in fixedness of the signals and in how likely they trigger certain responses in the audience that receive them. In cases where invariable signals precede invariable responses, there is little room for cognition. Therefore, dispensing of the cognitive ‘waystation’ in such cases does not represent a substantial loss, and communication can be viewed as signals or actions used to alter behavior. The breadth of communicative behavior, however, cannot be fully encompassed by fixed signals with involuntary responses.

Bypassing cognition becomes more difficult when the signals and responses are not fixed, but rather show some degree of variability. Flexibility in communication was first recognized by zoosemioticians studying the meaning of animal signals (e.g., Marler, 1961; Plooij and Lock, 1978), and later by researchers interested in intentional and goal-directed communication (e.g., Tomasello et al., 1985; Byrne et al., 2017). Both the early “signal meaning” approaches and the later intentional/goal-directed approaches to communication address cognitive aspects, but we will argue that neither of them are sufficient to fully explore how animals might use psychological states, and particularly some forms of inference about mental states, to communicate. In fact, some recent contributions that have embraced cognitive models of communication (e.g., Townsend et al., 2017) have flatly rejected mentalizing at any level and instead focus on superficial features of communication that denote flexible cognition. We think that this is a regressive mistake. The Gricean approach (Grice, 1957, 1969), which theorizes a high level of cognitive complexity, including pragmatic meaning, in communicative exchanges, is difficult to implement in investigations and interpretations of animal communication. The central idea that mentalizing plays a role in animal communication, however, deserves careful consideration.

One problem with completely rejecting mentalizing in animal communication, particularly if one is interested in the flexibility of a communicative system, is that mentalizing unlocks an unprecedented level of flexibility in human communication. Since many cognitive approaches to animal communication have used human communication as a point of comparison, particularly in considerations of the evolutionary origins of human language (e.g., Hewes et al., 1973; Zuberbühler, 2005; Scott-Phillips, 2015), it is at the very least questionable to *a priori* discard mentalizing. Although documenting flexibility in animal communication by means of behavioral indicators such as means-ends dissociations, contextual variation in signal use, and audience effects is a necessary first step (e.g., Tomasello and Zuberbühler, 2002; Tomasello, 2009 for review), such indicators explain neither the origin nor the psychological underpinnings of flexible responses. Producing a descriptive list of behavioral indicators of flexibility (goal-directedness) without digging deeper into the psychological process that give rise to those responses seems a missed opportunity. The problem is further compounded by the fact that referential and intentional communication are often used to explain language evolution (e.g., Arbib et al., 2008), but language is a system with mentalizing at its core (Grice, 1957, 1969; Wilson and Sperber, 2002). Without postulating some ability to make inferences about mental state to some forms of animal communication, the leap from animal to human communication, and language in particular, might be too great to be realistic. If the cognitive complexity of human communication is the measuring stick against which animal systems of communication are compared, at least in investigations of the evolutionary origins of language, then there exists a gap between the complexity of communicative behavior explained by the intentional model of communication and the ostensive-inferential models of human communication whose potential application has been discussed in certain animals, such as non-human primates (hereafter, “primates”).

In this article we propose a solution to this gap in current models’ explanatory power, for use in situations where the communicative behavior of a species or taxa involves an apparent level of flexibility and pragmatism not fully explained by existing models. We would like to introduce a model of communication – “inferential communication” – which we will distinguish from the model of intentional communication (e.g., Woodruff and Premack, 1979) and differentiate from other descriptions of inferential communication discussed by Fischer (2013); Fitch (2015) as well as those posited in developmental literature and studies of linguistics (e.g., Sperber and Wilson, 1986; Scott-Phillips, 2015; Moore, 2016). Our model is not intended encompass the same scope as global models of animal communication with ultimate explanations for communicative behavior. We submit inferential communication as a proximate model of communication which elaborates on ultimate explanations of communicative behavior by outlining some of the cognitive mechanisms that may operate within these ultimate models.

As we hope will become apparent, our proposal differs from cognitive models of animal communication that incorporate inferential processes on the one hand, and human ostensive communication on the other, along three main dimensions: the nature of the inference, the type of pragmatics involved and the role of informative intentions. We will combine the comparative research that has been accumulated in the last three decades on referential and intentional communication with data on social cognition and inferential reasoning to establish the theoretical foundations for our perspective on inferential communication. Thus, one of our key proposals is that mental state attribution, rather than being a problem, it is part of the solution. Together with inferential reasoning, it constitutes the cognitive substrate of flexible communication.

Our paper is organized as follows. First, we will summarize the traditional approaches to animal communication, in order of increasing engagement with cognition, and provide the theoretical background to contextualize the model we now propose. Second, we will outline the model of inferential communication, specifically with respect to primates, distinguishing our proposal from previous characterizations of inference in communication. Third, we will delineate the cognitive skills and mechanisms required for each increasingly mentalized level of complexity within our model. Fourth, we will shed light on the applications of inferential communication, from both a theoretical and experimental perspective, and explain the breadth of taxa to which it could potentially be applied. Finally, we will place inferential communication into the broader field of theoretical approaches to primate communication.

## From Signals to Intentional Gestures

### Manipulation Model

To appreciate the theoretical justification for inferential communication, it is critical to review both the tenets of ultimate approaches to animal communication and the questions they leave unanswered. The earliest ethological models of animal communication, including non-human primate communication, were founded in behavior, not cognition (see Table 1). Building on the work of Tinbergen (1952), Lorenz (1966), who created the foundation for phylogenetic preservation of evolutionarily successful behaviors, Dawkins and Krebs (1978), Krebs and Dawkins (1984) asserted that animal systems of communication are the result of repeated, non-communicative instrumental actions that become phylogenetically ritualized to prompt certain behavioral responses in others. Just as instrumental actions affect the environment to produce a certain result, communicative signals act on others to induce certain behaviors. If successful, the signaler will have incurred benefit as a result of the exchange, and thus the signal persists as a function of evolutionary fitness. This non-mentalized, behavior-centric approach is upheld in some modern work (e.g., Owren et al., 2010), where animal communication is described as an effort to influence the behavior of another and is placed in the shared evolutionary timeline of living primate species, including humans, as a necessary but distant step in the evolution of human language.

**TABLE 1:** Ethological models of communication including the origin and signal-referent relation as well as their key cognitive concepts.

Model	Sub-discipline	Signal origin	Signal-referent relation	Key cognitive concepts
Manipulation	Behavioral ecology	Innate	Fixed	n/a
Information	Zoosemiotics	Innate/learned	Flexible	Semantic signal encoding and decoding functional reference

If we apply this model to an example of a communicative interaction between two primates, the ritualized format of the exchange becomes clearer. In this example, one primate, Cindy wishes to be groomed by another primate, Louis. Accordingly, Cindy moves toward Louis and presents her shoulder, a behavioral pattern known to culminate in the receipt of grooming (Hobaiter and Byrne, 2014). Louis grooms Cindy’s shoulder, and Cindy therefore receives fitness benefits associated with grooming. Viewing this exchange through the lens of communication as manipulation, Cindy has engaged in a ritualized action which likely developed from the necessary instrumental actions associated with grooming, i.e., moving the body part close enough to allow grooming to occur. This action manipulated a response from Louis, the outcome of which benefited Cindy, who is therefore likely to repeat the gesture in the future, and the gesture is maintained, over evolutionary time, in this primate gestural repertoire. Notably, the ritualization of gestures here is from a phylogenetical perspective, not an individual one, and thus does not ascribe an individual representation or any cognitive process underlying the behavior to either party.

This model of communication offers an ultimate explanation of communication with broad taxonomic applicability; the same principles of manipulation and evolutionary fitness that explain the phylogenetic preservation of primate gestures explain the mating display of a bower bird or the aposematism of a toxic insect. This model does not, however, offer proximate explanations for the behavioral patterns of communication; it allows for situations where the induced response of the recipient is the result of *understanding* the manipulation and situations where the induced response is merely *a reaction to* the manipulation, the latter of which requires no cognitive engagement with, or even awareness of, the signaler’s desired outcome. There is an opportunity, therefore, for proximate models of communication to elaborate on the means-ends process of communication-as-manipulation by positing the mechanisms that might underlie the communicative behaviors.

### Information Model

Following Shannon and Weaver (1949), Marler (1961) proposed the theory of animal communication as information. This model characterizes information as the reduction of uncertainty on the part of the recipient, where the signaler encodes signals with informational meaning, and the recipient can decode these signals to access information. Although the informative signals are not necessarily under the intentional control of the signaler, they are still adaptive, just as in the manipulation model in the sense that they facilitate the desired outcome from the recipient. As a complement to the manipulation model, which more readily explains the fitness benefit of the signaler, the information model explains the adaptive benefit to the recipient more clearly – the recipient can achieve greater fitness by properly decoding the signal, gaining easier access to cooperative, affiliative exchanges, as well as easier interpretation of fearful, aggressive, or competitive displays.

Although the information model, which predates the manipulation model (see Dawkins and Krebs, 1978; Krebs and Dawkins, 1984), is still mainly centered on an ultimate perspective on communication, its principles eventually facilitated research on the cognitive mechanisms underlying communication. The process of giving and receiving informative signals can involve cognitive skills, including semantic encoding/decoding and functional reference (see Table 1). Furthermore, within this informational model, signals cannot necessarily be mapped 1-1 onto meanings, but may demand the use of contextual cues for accurate decoding (Smith, 1977).

Following our earlier example of an exchange between primates, the informational model of communication would interpret the actions *via* the route of informational transmission. Cindy wants to be groomed, and she encodes this information in a signal – a big, loud scratch across her own chest (Hobaiter and Byrne, 2014). As the scratching behavior is a non-instrumental signal, meaning that it does not act directly on the body of the recipient, Louis must decode this signal based on contextual cues and existing knowledge of the signal, and in doing so, receives the information that Cindy wants to be groomed. Louis may produce the desired behavior, or not, depending on the context and the fitness benefit to himself. Not only does this informational perspective address the success of the exchange from the perspective of both the signaler and the recipient, it also opens the door for an element of cognition: encoding and decoding of non-instrumental signals. Although not all informative signals require encoding and decoding – it is equally possible to inadvertently signal information and induce an innate reaction to that information – encoding and decoding become possible under this model of communication, which permits questions relating to cognitive engagement with the act of communication.

The informational perspective, though more robust in its mechanistic considerations, is more a behavioral model of communication than a cognitive one, and thus has theoretical limitations in its ability to fully characterize the cognitive abilities of certain species within communication. It describes cognitive engagement on the level of signal decoding and introduces the concept of flexible interpretation (i.e., varied interpretation of the same signal based on context). It does not, however, address the question of referentiality, at least, not in its earlier iterations (Seyfarth et al., 1980). Vocalizations or gestures encoded with information could be produced voluntarily or involuntarily, while still consisting of a non-instrumental signal encoded with valuable information for the recipient. Modern work within this paradigm (e.g., Tomasello and Zuberbühler, 2002; Leavens et al., 2004), asks this question of intentionality and referentiality, but does not conclusively conclude that the signaler or the recipient have an internal representation of the information, and rather, could be exhibiting “functional referentiality,” characterized by signals provoked directly by the external stimuli about which they contain information (Slocombe and Zuberbühler, 2005). Without an ability to account for internal representation of intention and meaning, the informational model of communication is inherently limited to basic, practicable cognitive mechanisms – encoding and decoding – which do not encompass the rich breadth of possible mentalizing in primate communication.

## Intentional Communication

Intentional communication, also known as goal-directed communication, the third and final historical model of communication, can be considered the first of three fully cognitive models (see Table 2). It introduced two critical cognitive skills – intentionality and goal-directed signals. Plooij and Lock (1978), Woodruff and Premack (1979) were among the first to thoroughly address the question of intentionality in animal communication, specifically in the communication system of primates. They characterized intentional communication as transmission of information between a signaler and a recipient adhering to three main criteria: first, the signaler must be aware the transmission of information will result from the signal; second, the signaler expects that the recipient will similarly be aware of the transmission of information; and finally, the signaler must be able to selectively control their own signals in order to transmit the desired information. Later work (e.g., Tomasello et al., 1985, 1989; Hopkins et al., 2007; Byrne et al., 2017) on intentional communication follows several core criteria for intentionality, first defined by Bretherton and Bates (1979) for use in developmental psychology. These core hallmarks of intentionality include attentional monitoring, gaze-alternation, persistence, and elaboration.

**TABLE 2:** Psychological models of animal communication including the signal origins, the signaler’s intention, the recipient’s decoding, and the cumulative requisite cognitive skills (later models include those of previous ones).

Model	Signal origin	Signaler’s intention	Recipient’s inference	Cognitive skills
Intentional Communication	Phylogenetic ritualization Ontogenetic ritualization	I want her to do X for me	n/a (I will do X to her)	Goal-directed signals Intentionality Referentiality Awareness of informational transmission
Inferential Communication	Inference	I want her to do X(= **x_1_ + x_2_ + x_3_**) for me	**What does** she want me to do to her**?**	Prosociality Informative intention
Ostensive Communication	Conventionalization Imitative learning	I want **to tell** her to do X for me	What does she want **to tell** me to do to her?	Communicative intention Recursive mental states/3rd- and 4th-order theory of mind

*A key aspect of inferential communication is that the signaler creates a new signal (or modifies an existing one) to instruct the recipient what to do. X(= **x_1_ + x_2_ + x_3_**) is meant to indicate that the signaler provides not just information about their goal, but also instruction about how to do a particular action. Bold lettering in the signaler and recipient column indicates the new component in each model compared to the previous one.*

While at least a subset of these criteria are necessary to indicate intentionality, they alone are not sufficient to conclusively demonstrate it. Townsend et al. (2017) note that, although there is no specific combination of criteria that would absolutely indicate intentionality, more indicators for any particular species or experiment serve as stronger evidence that the intentionality is genuine. Furthermore, we argue that intentionality is most likely to be at work when it is robust in the face of experimental perturbation. If flexible, apparently intentional communication cannot be transferred to a new situation where the old conditions of the successful communicative exchanges do not apply, and exchanges are unsuccessful in this new setting, then the communicative system may be more rigid than initially indicated by successful demonstration of the above criteria. Vail et al. (2013) demonstrated several attributes of intentional communication in coral reef fish (*Plectropomus pessuliferus marisrubri*), theoretically suggesting that intentionality may be more widespread than the complexity of the behavior might suggest. It is unknown, however, whether the apparently referential signals in fish would stand up under multiple, varied circumstances, which would be stronger evidence of flexible, goal-directed, intentional communication. If it was indeed the case that coral reef fish could successfully transfer this behavior to a new situation, then there would be no reason to deny the potential for intentional communication in their species. Each of the criteria for intentionality, including flexible transference of the intentionality to new circumstances, has been demonstrated, experimentally or observationally, in primates, particularly great apes (Leavens et al., 2005 for review; Graham et al., 2020).

**FIGURE 1:**
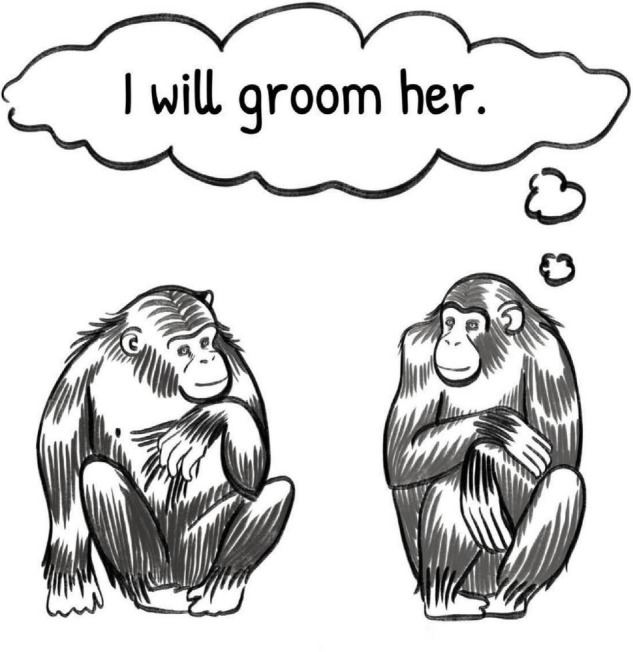
Illustration of two primates engaged in a communicative exchange depicting the recipient’s (lack of) inference under the intentional communication model. Illustration by Sadie Tenpas.

Carrying our primate grooming example forward, we now apply the intentional model of communication to these actions. Cindy, the signaler, must first open an attentional channel with Louis, the recipient, ensuring that she has his attention either through the use of an auditory or tactile “attention-getter” signal (Leavens et al., 2005), or by checking for existing visual contact. Cindy must have an internal representation of what she wants – grooming – and an awareness that she needs to transmit information about her goal – her desire for grooming – to Louis. She produces the signal, the big loud scratch from earlier, intentionally, and monitors Louis’ response, to determine whether the communication was sufficient to meet her internally represented goal. Louis, the recipient, must attend to Cindy, and must be aware that information is encoded in the signal, thus prompting him to decode it. As before, Louis can provide the desired grooming behavior, or not, at which point Cindy may persist and produce the gesture again, or she may elaborate, by producing a different signal which can also be decoded to request grooming (see Figure 1).

Cindy, in this example, is displaying new cognitive skills compared to those demanded by the previous models of communication. She is engaging in goal-directed communication, where she is internally motivated by her own goal and is using communication as a means of achieving it. She is displaying intentionality, wherein her actions are under her voluntary control, and, at this stage, she is communicating referentially, in that she is making direct, intentional reference to what she wants. Notably, the same cognitive mechanisms are not necessarily required of Louis, other than conscious awareness of the transmission of information. Although, according to this model of communication, he must be aware that there is information to decode, his response to that information does not necessarily need to be voluntary, for the communication to be successful. As in above examples, his response to the information he has decoded could be innate, or externally motivated by the stimulus of the information, rather than motivated by his own internal representation of Cindy’s goal.

Research using the framework of intentional communication has amassed a substantial body of evidence to support flexibility in primate communication (Liebal et al., 2014, for review). Regarding referentiality in primates, several studies have found evidence to support functional referentiality in the vocal domain (e.g., Slocombe and Zuberbühler, 2005, but see Fischer and Price, 2017 for an opposing view), and in the gestural domain (Call and Tomasello, 2007 for review). These are crucial findings for intentional models of communication, and they provide a framework within which to describe some of the flexible and behaviorally complex communication observed in primates from a cognitive standpoint. In our view, however, they still fall short of fully explaining the mechanisms at play in production and interpretation of communicative behavior in species with complex cognitive engagement during communicative acts. Intentional communication, as a model, invokes a means-ends dissociation, in that it describes observed behaviors in the context of their relevant psychological effects, but does not delve into the actual cognitive processes that permit these cognitive outcomes. It is clear that intentionality and flexibility place cognitive demands on both the signaler and the recipient, but the exact psychological processes are not illuminated. In fact, when we thought that the field was ripe to explore the psychological processes in greater detail, researchers have hesitated to take what we see as the next necessary step in unraveling the complexity of primate communication. In order to further advance our understanding of the cognitive mechanisms underpinning communication, we need to look beyond intentionality and toward psychological states. We propose the model of inferential communication as a means of explaining and investigating the cognitive, mentalistic aspects of communication, and to form a bridge between existing models of primate communication and the ostensive, language-oriented models found in the human developmental literature.

## The Model of Inferential Communication

As a theoretical model, inferential communication can be viewed as a system of conveying messages which operates outside the confines of codified, semantic gestures or vocalizations (Wilson, 1998), and which requires the integration of known information and context to interpret informational meaning. While we do not assert that inferential communication is engaged during all communicative interactions in any species, including humans, we submit this model as an explanatory and heuristic tool to investigate communicative behavior where inferential leaps of understanding, for both signalers and recipients, are required for successful transmission of information. When alternative explanations of apparently successful communicative behavior are ruled out, it allows for the investigation of higher-order cognitive mechanisms, such as mental state interpretation, prosociality, and, most crucially, *rational inference*. Crucially, in our model, inferential thinking is required of both the signaler, who must account for the leaps of understanding the recipient may make when deciding on the level of ambiguity in the signal, and the recipient, who must infer the meaning of the information being conveyed.

Many authors have noted there is ample evidence that recipients infer meaning from signals (Fischer, 2013; Fitch, 2015; Fischer and Price, 2017; Seyfarth and Cheney, 2017). However, the kind of inferred meaning that we endeavor to investigate differs from other proposals in terms of the type of inference that supports the communication and the type of pragmatics involved. First, we use inference more narrowly than other authors, to distinguish it from other processes. In a broad sense, when a baboon hears the call of his consort behind some bushes, he may infer that she is located behind those bushes (Fischer, 2013). But it is also possible that the individual has learned over time that when that call is produced, a particular female will appear behind those bushes – so an association rather than an inference might be doing the work of deciphering the signal. Another interpretation of “inference” refers the integration of information from multiple sources to make a decision (Fitch, 2015; Fischer and Price, 2017). There is no doubt that integration – putting together disparate pieces of information - is a fundamental aspect of inference (Tolman, 1932; Premack and Premack, 1994). But integration can also be achieved by processes such as conditional discrimination. When a baboon hears a specific female’s call, but he also sees that her juvenile offspring are nearby, he may respond differently to her call than if they were absent, not because he has inferred different meaning from her signal, but because he has learned over repeated exposure to similar situations that the appropriate response differs from a situation in which he is alone with the female. In this case, he is not exhibiting inference, but merely learned different responses to different contexts. We agree that inference requires the integration and assimilation of multiple pieces of information to guess at outcomes (i.e., “contextual pragmatics” in Fischer and Price, 2017). But additionally, inference requires that this integration occur in a novel situation, not one that has been encountered before (see section “Practical Applications of Inferential Communication” for an example of how to study this form of inference). Furthermore, our definition of inference affords inferential thinking to the signaler, which allows a greater depth of cognitive engagement, including intended meaning from the signaler.

Second, there is no question that contextual pragmatics play a crucial role in the inferences recipients make in communicative exchanges. For instance, baboons may use the time of the day, the location, the activity or even the reproductive state of their groupmates to derive meaning from signals (Fischer, 2013; Fischer and Price, 2017). In our model of inferential communication, however, we open the door to mental state attribution and even the notion of common ground. We do not ascribe the most elaborate forms of mental state attribution and common ground to the inferences made in our model but propose that more basic levels of mental state attribution, such as knowledge state and past shared experiences, may be taken into account by both parties. This constitutes at least an entry point into a dimension that escapes contextual pragmatics, thus potentially bringing communicative exchanges closer to linguistic pragmatics. Note that our goal is not to downplay the importance of context in deriving meaning. On the contrary, contextual pragmatics play a fundamental role in the communicative exchanges of humans and primates (and possibly other animals), but we argue that there might be more to inference within animal communication than just contextual pragmatics, at least in certain interactions.

We also differentiate our model of inferential communication from the models of ostensive communication (Scott-Phillips, 2015; Moore, 2017; Heintz and Scott-Phillips, 2022), particularly with respect to the nature of inference and the depth of mental state attribution. Models of ostensive communication highlight the importance of inference in communicative exchanges, but they use inference in a much broader sense than we do in our model. Ostensive models also emphasize the role of complex mental state attribution, often articulated as informative and communicative intentions. We discuss and contrast these models with our own proposal in greater detail in Section “Beyond Inferential Communication: Ostensive Communication.” For now suffice to say that we conceive inferential communication as the vital missing link between models of intentional and ostensive communication.

One of the main virtues of intentional communication is that it places flexibility and individual use of signals center-stage. However, the flexibility afforded by this model is rather limited. The origin of signals in intentional communication is either phylogenetic or ontogenetic ritualization. Phylogenetic ritualization produces species-specific signals potentially shared by all members of a species (and other closely related species). Signals *per se* are rather fixed, although their usage can show some flexibility, particularly in the gestural domain, in terms of when individuals choose to produce them, and whether they repeat them or replace with other signals in their repertoire when they fail (Liebal et al., 2014; Tomasello and Call, 2019 for review). This certainly shows some voluntary control over signals, but phylogenetic ritualization cannot produce new signals within an individual’s lifetime. This is mainly the task of ontogenetic ritualization whereby two individual shape each other’s behavior over repeated interactions so that they transform instrumental into communicative actions (Pika et al., 2005).

The production of novel signals is an important achievement, but ontogenetic ritualization is a slow process likely governed by associative learning. This means that new signals invariably require repeated interactions before they become fully functional. Attempts to document other forms of learning, most notably imitative learning, have failed to produce convincing evidence this form of learning is responsible for gesture acquisition in chimpanzees (Tomasello et al., 1997; Tennie et al., 2012). Inferential processes offer an alternative to ontogenetic ritualization and associative learning so that individuals can spontaneously invent gestures that others might be able to comprehend. Inference has been documented in numerous studies of physical cognition in primates (e.g., Hill et al., 2011; Petit et al., 2015; Völter and Call, 2017). Whether primates can also use inference in communicative situations is unclear but worth investigating. Table 3 presents the types of inference that could be involved in primate communication. Each of these types requires increasing levels of cognitive sophistication. In the subsequent sections, we develop our proposal for inferential communication starting with situations involving social inferences in the absence of communication.

**TABLE 3:** Social inference (non-communicative) and three types of inferential communication presented in ascending order of complexity in terms of the signal production and comprehension.

	Concept	Signaler’s intention	Recipient’s inference	Cognitive skills
Social Inference	Instrumental Action	I want to do X	What does she want to do?	Goal attribution
	Ambiguous Signal			Prosociality Informative Intention
Inferential Communication	Re-purposed Signal	I want **her** to do X (= **x_1_ + x_2_ + x_3_**) **for me**	What does she want **me** to do?	Innovation Context Rationalization
	New Signal			Iconicity Pantomime

*Also depicted is the signaler’s intention and recipient’s understanding of those signals in reference to the intention communicated by the signaler. Bold lettering represents the social and goal-directed nature of the signaler’s intention and the recipient’s inference.*

### Social Inference

Of all the cognitive skills included in the model of inferential communication, the capacity for inference is both the most obvious and the most critical. Inferential communication is a system which demands a certain flexibility in interpretation of social interactions, where individuals must make leaps of understanding regarding the social behavior of another actor. One might call this “social inference,” defined here as a situational understanding of another’s actions beyond the available semantic information. Not restricted to communication, this ability includes successful interpretation of another’s goals, intentions, or desires, in both cooperative and competitive contexts. Although social inference is not necessarily within the realm of communication, it is a vital prerequisite to inferential interpretation of another’s communicative behavior. Social inference asks, “What does she want to do?” an open-ended question that relies on behavior, context, and inference in order to successfully attribute the ultimate goal to a set of actions performed by another.

Take, for example, our grooming primates. Now, rather than describing a communicative exchange, we can use their behavior to illustrate social inference. In this situation, Cindy grooms herself, producing species-typical grooming behaviors, such as plucking and licking certain areas of the body. She does not specifically intend to produce any particular signal, but she is observed by Louis, who makes inferences about her goals. Louis, observing her plucking behavior, could mentally represent her goal, which might be to alleviate an itch, clean a wound, or even to self-soothe after a tense encounter. Louis’ inferential interpretation could be based on contextual cues (e.g., a visible wound, having witnessed a fight between Cindy and another individual, etc.), and/or past experience (Louis has groomed himself in the past and is aware of the benefits). Louis’ capacity for inference, demonstrated here in his differential interpretation of Cindy’s actions based on context, invokes the cognitive skill of goal attribution, which is not a requirement for the recipient in any of the previous models of communication. Additionally, Louis shows evidence of addressee awareness, in the sense that he is aware that he is not being addressed, which invites a different interpretation of Cindy’s goal than if the behavior had been communicative and directed at him.

There is ample evidence for social inference in primates, including rational imitation, where great apes were less likely than human children to perform extraneous actions to complete a task, even when those actions had been demonstrated by a human actor (Call and Tomasello, 1998; Buttelmann et al., 2007, 2008). The apes appeared to infer the ultimate goal of the experimenter’s actions and were able to produce a different, streamlined set of actions toward the same goal, rather than copying the experimenter’s exact movements, indicating that they were able to use the experimenter’s behavior to form a representation of their intentions. While perhaps reflecting less of an inclination toward social learning than human children, who readily imitated both the necessary and extraneous actions of the experimenter, these studies demonstrated that apes were able to infer the ultimate goal of the human’s task, and thus eliminate unnecessary steps, suggesting a successful leap in understanding regarding the human’s ultimate intention. Great apes also flexibly interpreted an experimenter’s behavior in differing contexts, although the experimenter’s actions were identical in both situations. Subjects were more likely to select one of two boxes when the experimenter “intentionally” dropped a marker on it versus when they “accidentally” dropped a marker on it (Call and Tomasello, 1998), which required inferences about the experimenter’s goal when dropping the marker. Similarly, apes differentially adjusted their waiting behavior when experimenters were performing necessary actions on a puzzle box to retrieve food, compared with contexts where those same actions were superfluous, suggesting that they made inferences about the goal of those actions based on different contexts (Buttelmann et al., 2012). In each of these examples, despite identical semantic information, apes flexibly adjusted their responses (e.g., selection behaviors, waiting behaviors, begging behaviors) in response to different perceived goals from the experimenter. This evidence suggests that great apes have the ability to make pragmatic inferences about social behavior based on clues from context alone.

Evidence of social inference in primates is not limited to the interpretation side of social interactions. When it comes to production, both apes and monkeys show flexible adjustment of vocal signals based on the identity of the recipient (Cheney and Seyfarth, 2018). For example, chimpanzees produce food grunts toward “friends” more often than “non-friends” (Schel et al., 2013), and female baboons have been shown to selectively produce conciliatory grunts, mediated by the likelihood that the recipient will view their behavior as affiliative, where immediate past experience and long-term dominance dynamics appear to be the moderating factor (Cheney and Seyfarth, 2008). Audience effects such as these have been noted as evidence in reviews of intentional communication in primates (Liebal et al., 2006; Byrne et al., 2017), but they also present a potential case for inferential cognition, if and when these signals are voluntarily produced or withheld. While changes to vocal signals according to varying situational context (Seyfarth and Cheney, 2010 for review) are not enough to suggest social inference, variation in communication behavior regulated by *social* context, combined with voluntary control of these signals, allows that signalers may have an awareness that the intended message may be received differently by different individuals, depending on the existing social relationship with the specific partner. The possibility that primates can flexibly adjust communication behavior based on the varying potential outcomes from different recipients suggests that they have may be able to base these decisions on inferences from past social experiences, which goes beyond the realm of mere intentionality.

### Inferences Using Communicative Signals

#### Ambiguous Signals

In the case of fixed, semantic, unambiguous signals, advanced cognitive mechanisms are not necessarily required. In the case of flexible, ambiguous signals – those which are used in multiple contexts to mean different things – inference is a necessary component of interpretation. In order to apply social inference to the realm of communication, we must first consider the mentalized question at hand, for both the signaler and the recipient. The signaler asks, *“What do I want* ***him to do****?”* This question involves both an informative intention (that which she wants him to do), and a prosocial desire (the fact that she wants or needs him to do it, at no immediate benefit to himself). The recipient, on the other hand, asks, *“What does she want* ***me*** *to do?”* This question has an inherently prosocial component, which denotes a new cognitive skill, not explicitly required by past models, on the part of the recipient.

**FIGURE 2:**
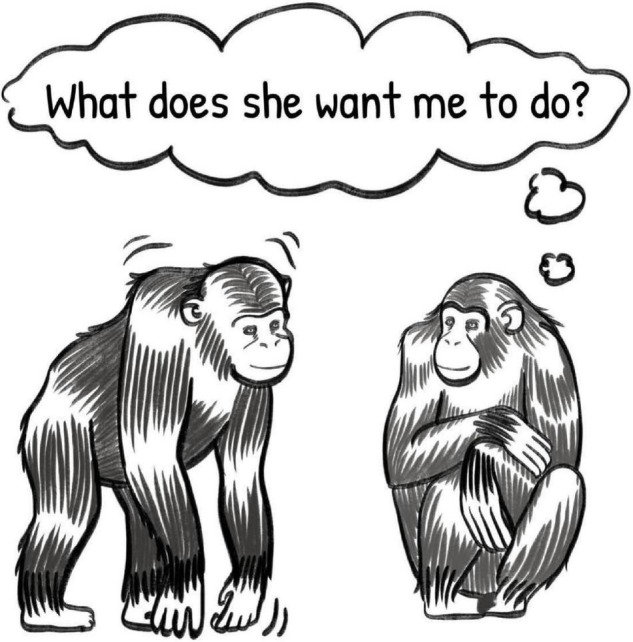
Illustration of two primates engaged in a communicative exchange using ambiguous signals, depicting the recipient’s inference under the inferential communication model. Illustration by Sadie Tenpas.

This arrangement requires the signaler to transmit a message which relies on the recipient’s capacity for inference in order to be fully interpreted. She must establish attentional contact with the recipient to open a channel of communication between them, and must request help, providing instructions as to the nature of the help required. If she uses an ambiguous signal to provide instructions, however, the recipient must use inference to successfully interpret the message. For example, returning to Cindy and Louis, we now imagine a situation in which Cindy wishes to be groomed by Louis. She faces Louis, ensuring that he observes her, which opens the channel of communication. She taps her knuckles against the ground and bobs her body up and down, a gesture which is commonly used to initiate play, but has also been observed preceding grooming (Tomasello et al., 1997). In this gesture, she has both requested help from Louis, and provided instructions – she wants help in the form of grooming. She has, however, relied on her knowledge of the contextual inferences Louis is most likely to make, as well as the belief that Louis will be sufficiently motivated to help her. Louis, for his part, must attend to Cindy, be motivated to help, and understand the instructions, using context to disambiguate her gesture. If the communication is successful, then Louis will use contextual cues (for example, past experience with Cindy, in which they have rarely engaged in play) to interpret her instructions, and he will infer that she wants to be groomed (see Figure 2).

Regarding flexible interpretation of ambiguous behavior, there is evidence that primates are capable of such inferences. For example, great apes successfully differentiated between the same action from a human experimenter, producing more begging and impatience behaviors when the experimenter’s action could be interpreted as “unwilling” to provide food versus “unable” to provide food (Call et al., 2004). From the production side, apes were able to modify the shape and location of their pointing behavior when their options were arranged such that a simple forward point would be ambiguous, suggesting that they recognized the need to disambiguate their pointing for the experimenter’s successful comprehension and that they were able to apply that understanding to their actual gestures (Tauzin et al., 2020). Furthermore, great apes will monitor the success of a relatively ambiguous signal (e.g., begging), and elaborate with different, additional gestures (e.g., pointing at the desired option), if the desired outcome is not achieved (Leavens et al., 2005). This demonstrates a willingness to produce ambiguous signals, suggesting that the apes have some expectation that the signals will be successfully disambiguated by the experimenter, and also the capacity to choose whether or not to be more specific, at potentially higher cognitive cost to the signaler, if the ambiguous signal fails.

#### Re-purposed Signals

In situations where an ambiguous signal made unambiguous through inferential interpretation is not sufficient to thoroughly instruct the recipient, the signaler may turn to other resources to produce an instructive signal. One possible approach is to use an existing signal within the communicative repertoire, but in a brand-new context, relying on the inferential capability of the recipient to interpret the familiar signal in a new way. The situational question remains the same for both the signaler and the recipient – “What do I want him to do?/What does she want me to do?” – but new cognitive skills are required at this level of complexity. In addition to the required capacity for inference and prosocial behavior, the signaler and the recipient must both take a creative leap and rationalize the otherwise nonsensical use of the signal in the current situation, giving it new meaning.

**FIGURE 3:**
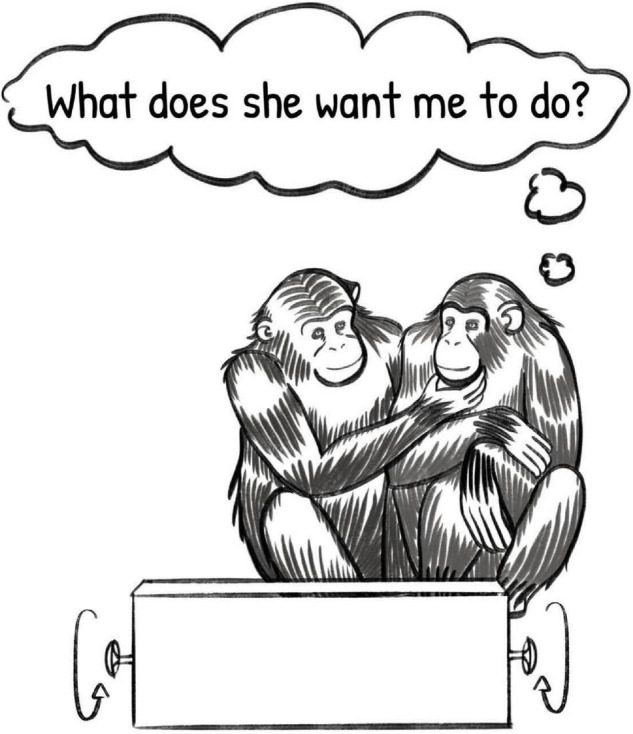
Illustration of two primates engaged in a communicative exchange using re-purposed signals, depicting the recipient’s inference under the inferential communication model. Illustration by Sadie Tenpas.

If we follow primates Cindy and Louis into a new situation, an experimental setting in which they must work together to open a puzzle box, we can hypothesize an interaction using this form of inferential communication. Cindy wants Louis to help her open a locked puzzle box, which can be achieved by turning two wheels, simultaneously, at opposite ends of the box. As neither can reach both wheels, they must coordinate to solve this problem. As before, Cindy must establish a communicative channel with Louis, by looking at him and ensuring that he sees her. As no fixed, semantic gesture exists in their shared repertoire to communicate “help me open this box,” Cindy produces a gesture more typically used to beg for food, a mouth stroke (Tomasello et al., 1997). The gesture is nonsensical in this context, as Louis has no food to offer her. Instead, Cindy has engaged in creative use of this gesture to encourage Louis to open the box with her. If this exchange were to be successful, Louis would correctly infer that Cindy does not want to share food, rather, he would rationalize the otherwise pointless gesture to a new meaning, and if sufficiently motivated, help Cindy open the box (see Figure 3).

This exchange relies on creative re-purposing of existing gestures, a capacity which has not been conclusively demonstrated in primates, but which can be hypothetically proposed in the model of inferential communication. Armed with this framework, it is possible to design experiments which more specifically demand this ability, to explore the cognitive skill and its presence or absence in primates.

#### New Signals

In its final possible level of cognitive complexity, inferential communication provides a platform for two actors to create an entirely new signal, rationalized and understood by both purely based on the context and their own capacity for inference. Still adhering to the question, “What do I want him to do?” the signaler instructs the recipient using an iconic gesture – one that does not exist in the known repertoire of the individual, and which pantomimes the action she is requesting that the recipient perform. This iteration of inferential communication adds two specific cognitive skills not required for earlier levels: iconicity and pantomime, which are necessary for both the signaler and recipient.

**FIGURE 4:**
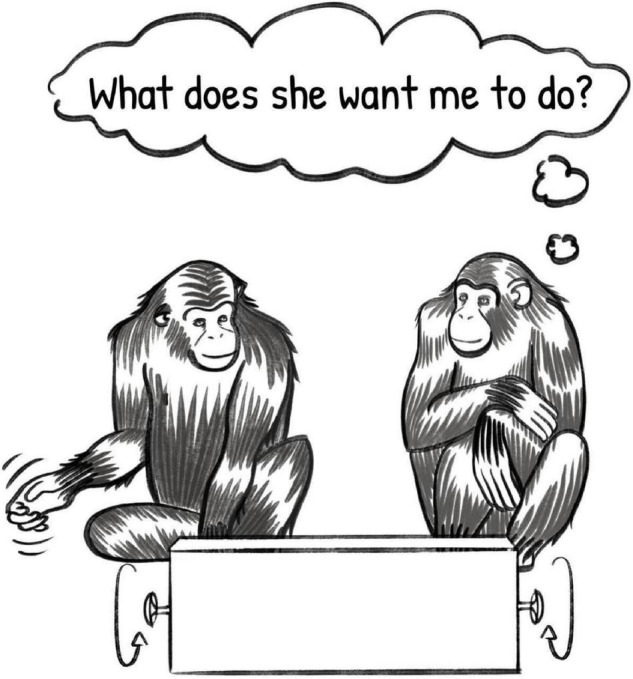
Illustration of two primates engaged in a communicative exchange using newly invented signals, depicting the recipient’s inference under the inferential communication model. Illustration by Sadie Tenpas.

If we return to Cindy, Louis, and the puzzle box, we can imagine a situation in which Cindy establishes that she has Louis’ attention, and then turns her hand in the air, miming the turning of the wheels on the puzzle box. Louis, observing this pantomime, interprets the gesture as an iconic representation of the desired action, understands Cindy’s request for help and the instructions she has given, and helps her open the box (see Figure 4).

While this type of interaction has not been systematically documented in primates, and it is unlikely that this type of interaction is common, preferred, or cognitively efficient for non-human animals, the question remains as to whether primates could exhibit these cognitive abilities if there were no other way to solve the problem. There is some anecdotal evidence that primates are capable of the two new cognitive skills seen here – iconicity and pantomime. Grosse et al. (2015) found that one chimpanzee, who had been partially reared by humans, engaged in an iconic gesture when a human experimenter required instruction to operate an apparatus. Additionally, great apes have been anecdotally observed engaging in pretend play, with or without the assistance of objects, suggesting some possibility of iconic representation of objects (Gómez, 2005). On the comprehension side of these abilities, great apes have been shown to learn locations associated with iconic gestures faster than locations associated with arbitrary gestures, suggesting that they have some ability to link the iconic nature of those gestures to their representational meaning (Bohn et al., 2016a). It is noteworthy that production of iconic signals, iconic play, and imitation of pantomimed gestures can be scaffolded with the support of physical objects, removing the requirement of intransitivity that is intrinsic to true pantomime (e.g., Call, 2001; Gómez, 2005; Tennie et al., 2012). This suggests that iconicity and pantomime are challenging cognitive skills for great apes and would require substantial prosocial motivation or necessity in order to be a cognitively efficient mechanism. Whether these anecdotal cases could be transformed into robust evidence of these cognitive skills in primates, especially in the absence of scaffolding, is unknown, but the question itself presents an exciting example of the investigations and experiments that become possible on the tails of inferential communication. It also invites the question of prosociality in primates, given that prosocial action is a critical component of the success of any inferential communication exchange, but especially those with increasingly difficult cognitive demands.

### Prosocial Motivation

One of the basic tenets of human communication is that it is a prosocial and cooperative enterprise (Hare, 2017). Although prosocial motivation can foster communicative exchanges, and it may be needed for language acquisition, we argue that it is not necessary for inferential communication because effective exchanges can occur even in the absence truly prosocial (i.e., altruistic) behavior. Apparently prosocial actions, required of both actors in inferential communication exchanges, can be understood with several different motivations in mind. On the surface, prosociality is defined as any action, whether requested or performed, that one actor completes for the benefit of another, with either no benefit or actual cost to themselves (Cronin, 2012). In practice, however, apparently prosocial actions, those performed at cost of one individual and benefit of another, may have motivations other than pure altruism. An individual could be motivated to behave in a prosocial manner due to a cost-benefit analysis, in which the continuing annoyance or harassment from the requesting individual is a greater cost than performing the action, and the actor is therefore sufficiently prosocially, if not altruistically, motivated. Alternatively, the actor could incur hidden ultimate benefits, such as augmented reputation, hopes for reciprocation, or, in humans, a proximate benefit of internal moral good feeling, which offset the apparent cost of the prosocial action. Thus, when we argue that prosociality is a requirement for successful inferential communication, we refer to the broad spectrum of motivations that could lead to apparently prosocial action. Apparently prosocial actions, whether altruistic or otherwise, have been observed in several species of primate, from tamarins (Cronin et al., 2010) to macaques (Massen et al., 2010), and to great apes (Pelé et al., 2009).

Altruistic prosocial motivation and willingness to engage in the inherently cooperative act of communication become more imperative as the cognitive load of the exchange increases. For the both the signaler and the recipient in a communicative exchange, the cost-benefit analysis of the effort to produce or interpret a communicative signal changes depending on the difficulty associated with interpreting the message. For fixed signals and ambiguous but commonly used signals, the cognitive effort may not override the beneficial outcome of the signaler and the potential hidden benefits for the recipient. When the more demanding cognitive skills mentioned above – creativity, rationalizing re-purposed signals, iconicity, and pantomime – are necessary for the exchange, the outcome must be more beneficial for both parties. Particularly for the recipient, it may be that this type of cognitive load is only worth the effort if the individual is truly altruistically motivated, a phenomenon which remains debated in primates (Cronin, 2012). Perhaps prosocial motivation is crucial to decode opaque messages that otherwise are simply not understood and consequently, ignored. It is possible, therefore, that lack of existing evidence for these later levels of inferential communication is caused by twofold limitations. First, the necessity for truly altruistic motivations, which appear to occur sparsely, if at all, in primates, and second, the difficulty of the cognitive mechanisms at play. It is possible, however, that in the presence of sufficient prosocial motivation, primates could produce and interpret these types of cognitively complex signals.

## Beyond Inferential Communication: Ostensive Communication

Although our focus is on inferential communication, it is critical to discuss ostensive communication for the sake of contrast and completion. Ostensive communication makes the leap from social inferences to communicative inferences – specifically, inferences about communicative intention (see Table 2). Communicative intention is traditionally understood as a mental state function, in which the communicator not only knows the mental state of the recipient, but consciously intends to manipulate that mental state by making their own informative intention manifest. This is combined with the recipient’s recognition that the communicator has an informative intention, which prompts the recipient *via* the presumption of relevance to make inferences about the meaning of the message based on contextual cues and mental states (Scott-Phillips, 2015). The capacity of primates to produce and comprehend communicative intention under this mentalistic definition is not clearly understood – it has yet to be conclusively observed or experimentally demonstrated in great apes, and it is seldom investigated in monkeys and prosimians (Moore, 2016). Some researchers assert that this cognitive capacity is unlikely to exist in primates, given the sufficiency of a sparser, more goal-directed and intentional model of communication to explain most communicative exchanges primates (Fischer and Price, 2017), and given that primates frequently fail tasks which require production or comprehension of communicative intention (Tomasello, 2008). This mentalistic definition of communicative intention requires recursive mental state attribution, including fourth-order theory of mind (Scott-Phillips, 2014), which many regard as too complex for primates.

Ostensive communication asks the question, *“What does she want* ***to tell me*** *to do?”* where not only the informative intention, but also the communicative intention, is manifest to the recipient. In our hypothetical primate example, Cindy wishes to be groomed by Louis. In order to accomplish this, Cindy makes inferences about Louis’ current mental state – his willingness to groom her, his awareness that she wants to be groomed, and their existing shared knowledge – and knowingly and intentionally sets out to alter his mental state with her message, such that he becomes aware that she wants to be groomed and is motivated to do so. Louis, likewise, as the recipient, must be aware that Cindy is attempting to alter his mental state, and uses that knowledge to make inferences about what she wants, based on the situation context.

Our model of inferential communication shares two key aspects with the model of ostensive communication: inference and mental state attribution. However, it differs in the type of inferences that it uses, and it lacks the most complex forms of mental attribution, particularly communicative intentions. Models of ostensive communication use inference with two meanings, one very broad (all communication involves some form of inference) and one rather narrow. For instance, Scott-Phillips (2015) describes inference as the interpretation of meaning based on evidence of informative and communicative intentions of the signaler. In our model of inferential communication, we do not ascribe expression or interpretation of communicative intention to either actor, but rather suggest that the signaler is relying on the recipient to make inferences about their goals (i.e., informative intention), rather than their communicative act itself. Thus, although we agree with Scott-Phillips (2015), Heintz and Scott-Phillips (2022) that communicative intentions may be beyond the capabilities of non-human animals, we argue that informative intentions might not be – signalers can express their goals informatively, but do not make their intentions manifest and recipients do not necessarily use presumption of relevance to infer meaning.

Recently, Heintz and Scott-Phillips (2022) distinguished between ‘intentional expression’ defined as the expression of mental states (e.g., a signaler may indicate what she wants to a recipient) and ostensive communication that requires making informative intentions manifest. We view intentional expression as similar (if not equivalent) to what we are calling inferential communication, except that we argue that informative intentions (perhaps in a more rudimentary form) are conveyed by signalers, but communicative intentions are not. Other authors have argued that non-human animals may even exhibit some forms of communicative intention. Moore (2017) argues that primates may indeed exhibit a form of Gricean, ostensive-inferential communication, but emphasizes the role of awareness of address on the part of the recipient to provide the context for interpretation, which is followed by inferences about the goal of the signaler.

The complexity of mental state attribution required by full-blown (human) ostensive communication is not yet evidenced in primates. Like the more complex levels of inferential communication, it is possible that both the cognitive and the prosocial demands are too great for the majority of communicative exchanges between primates. Perhaps, with evidence taken from an inferential communication framework, it might be possible, in the near future, to design experiments which better establish the limits of primate mental state attribution, to further bridge the gap between language-oriented developmental literature, with rich, Gricean interpretations of communication, and comparative literature, where interpretations are currently limited to description and suggestion of cognitive engagement. The model of inferential communication, when applied to observations and experiments in non-human animal behavior, presents the opportunity to ask theoretical questions about flexible communicative problem solving, theory of mind, and communicative intention.

## Practical Applications of Inferential Communication

Any newly proposed theoretical framework to study animal communication, has to consider its practical applications: what can this model offer, how can it be examined empirically, and which species are more likely to display it? In this section, we highlight some of the specific applications of inferential communication and propose some examples of experimental designs to test whether recipients, but also signalers, use inference in their communicative exchanges. To do so, we present three tasks, one using vocal communication and focused on inferred meaning, and the two others using gestures and focused on intended meaning. We intentionally provide methods examining both vocal and gestural communication in order to illustrate the complementary roles played by vocal and gestural communication in elucidating the intended and inferred meaning of ambiguous, re-purposed and novel signals. We close this section by outlining our criteria for determining whether a species might be a good candidate to investigate inferential communication.

A key application of inferential communication is to explain the origins of some gestures. It is recognized that gestures can arise *via* phylogenetic or ontogenetic ritualization (Cartmill and Hobaiter, 2019). Another mainly discarded form is third-person imitation (Tomasello et al., 1997; Tennie et al., 2009), although language trained apes have been reported to learn some signs by imitation (Fouts, 1972; Gardner et al., 1989). We propose that inference could serve as a fourth form of gesture acquisition; creating a new gesture to indicate old or new meaning, or less demanding, re-purposing a gesture, and here the work is in using it with a different meaning and especially interpreting it. Different from phylogenetic ritualization, where a successful gesture is preserved and inherited in the innate repertoire, and ontogenetic ritualization, where a gesture develops from repeated use of action-oriented movements, inferential development could explain gestures which originate as iconic or re-purposed movements and are practiced and used until they are semantically established between two or more individuals. Notably, this understanding of the origin of gestures would differentiate between ritualized gestures which iconically evoke the requested action, but evolve from the actual occurrence of the action, and inferred gestures, which originate from an iconic, pantomimed representation of the action.

It is crucial that inferential communication, as an origin of gestures and as a cognitive process, be explored experimentally. Novelty is an essential part of the development of new gestures; if two individuals use a gesture repeatedly, there is no need to invoke inference. Inference need not necessarily be applied in all communicative exchanges, but when the system is perturbed (new conditions or old conditions no longer apply) it can play a crucial role in the success of communication. Once invented by inference, a gesture may be used repeatedly, which can quickly mask its origins. Thus, experiments are critical in order to observe the emergence of new forms of communication.

In designing experiments to test inferential communication, it is essential to ensure that the task requires true inference – the integration of known information to understand a new scenario. Likewise, the experiment should require the use of pragmatic information on the part of both parties, not just on the order of situational context clues, but on the mentalized level of knowledge state, private interaction history, or individual preferences. Our first proposed experiment applies these two criteria to interpreting ambiguous vocal signals. We imagine an experimental setting in which the recipient of the communication knows two established pieces of information, which may have been learned by past inference, association, or simply occurred as a result of the individual’s maturation. The point is that the origin of the two pieces of information is not so relevant in our example. First, the recipient, a primate in this example, must be familiar with the species-specific vocalizations that individuals produce when they discover a cache of highly preferred food. Second, the recipient must be familiar with the individual food preferences of a particular groupmate. For instance, Cindy, our recipient, knows that Louis likes bananas but does not like grapes. This is something that she could have learned by observing Louis’ feeding patterns: always eating bananas with gusto but ignoring grapes, when both are available, and even when bananas are gone, Louis shows no interest in grapes still available. Cindy is also familiar with their species-specific food calls, which are associated not with a particular type of food, but with the discovery, prior to, but not during eating, of a cache of food.

In the test condition, Cindy is shown that one of two foods is hidden behind a bush, but she is not shown which type of food. Louis then appears and produces a food call upon encountering the food cache. If Cindy is indeed capable of integrating multiple pieces of known information to infer meaning in this new situation, we predict that she should infer that there are bananas behind the bush. Moreover, she should be surprised, in this instance, to search and discover grapes behind the bush, and this response pattern should be reversed if the caller was an individual who likes grapes and does not like bananas. Notably, experiments like this allows examination of the first exposure to this novel situation, which is important for evaluating inference. Associative processes require at least one event for learning to occur, which means the recipient’s reaction on initial exposure in the proposed experiment is a measure of true inference. As far as we know, this proposed experiment has not been done (but see Shorland, 2018 for a similar experimental paradigm), but we already know that chimpanzees integrate the food preferences of others and their visual access when choosing between two experiments – selecting the one which will give them the most favorable outcome (Eckert et al., 2018). This experiment would test whether they could extend this ability to integrate information to inference in communicative exchanges.

Compared to the work investigating recipient comprehension, much less has been done examining the inferential abilities of signalers, with some authors arguing that primate signalers do not intend meaning, recipients just infer it (e.g., Fischer, 2013; Fischer and Price, 2017). This is a sensible proposition given that primate vocal signals are fixed, apart from variation in the timing and context of their use. Such inflexibility in vocal production may not permit primate signalers much opportunity to imbue meaning to their signals. Gestures, on the other hand, are quite different in terms of their production. Gestures are grounded in bodily action; they are much more flexible than vocalizations, which opens the door for flexible variation that changes the potential interpretation of the signal. This flexibility also permits the creation of novel signals or the re-purposing of old signals to a novel use. Therefore, we challenge the idea that primate signalers in general do not ever intend meaning and argue that this conclusion may have resulted from asking this question from the perspective of vocal communication only.

The literature already contains some studies illustrating this point – examples we would argue indicate that signalers communicate intended meaning. For instance, Bohn et al. (2016b) found that great apes used a pointing gesture in an unusual way (pointing to an empty dish) to request food that was no longer in that dish. Pointing to an empty dish is atypical for apes, especially given that another dish containing food that was less preferred, but otherwise perfectly acceptable (they always ate this food in control trials), was present. Special care was taken to avoid training the apes to point to an empty container in the pre-test, where they witnessed that the experimenter got up as soon as the food was depleted, left the room, and brought in more food, without giving the subject a chance to point to the empty container. Importantly, apes only used this unusual gesture with an experimenter who had brought food in the past as soon as food had been depleted but not with an experimenter who had given them food but not brought it in the first place.

When we analyze the key features of this case, we conclude that pointing to an empty container qualifies as re-purposing a familiar gesture to communicate about an absent referent. First, pointing with extended fingers, unlike vocalizations, is not a species-specific gesture, but one that is acquired in contact with humans, thus showing some degree of flexibility in gestural acquisition. Second, the pointing gesture is directed at referents (e.g., food item) that are present (even when they are hidden), not to empty dishes, which suggests that the apes in the experiment were using the gesture in a novel way. Third, apes used the pointing to the empty container only with the experimenter that they had experienced bringing food, and not with others, suggesting that they accounted for the private interaction history between themselves and the experimenters in order to inform their knowledge of whether the gesture to the absent entity would be meaningful. There are other examples in which apes communicated what they wanted by re-purposing an action to request help from an experimenter. The bonobo Kanzi pounded on a nut to request that an experimenter to crack it open (Savage-Rumbaugh et al., 1986). In anecdotal observations, juvenile gorillas physically guided human researchers toward locked doors, using gaze-alternation throughout the movements, presumably to indicate to human observers what needed to be done (Gómez, 1990). The fact that in these instances apes established eye contact with the human experimenter when performing their actions toward the door and reduced their rate of these door-approaching actions when the experimenter left the room strongly suggests that the apes were using those acts to communicate with the experimenter, and not purely as a goal-oriented mechanism.

Intended meaning could also theoretically occur in more complex forms of communication, whereby apes invent a new gesture by, for example, pantomiming an action to indicate the tool that they require to obtain food. Yamamoto et al. (2012) reported that chimpanzees transferred tools to their partners following requests. Signalers used a hand begging gesture and recipients, who could see the kind of tasks that signalers were facing, selected the correct tool from an assortment of various tools and gave them to the signaler. When the recipient’s view of the signaler’s task was blocked, however, they handed tools randomly. This means that the begging gesture itself did not carry meaning about the type of tool. Context provided that information because the recipient could see the tool that was needed. Thus, the burden of decoding the message fell on the recipient who used contextual cues (the type of apparatus present) to infer meaning. This level of inference is based on contextual pragmatics, not mental states, but it begs the question: would the signaler become more specific in her request, and perhaps even invent a novel gesture by pantomiming the use of a specific tool, if the lack of contextual information persisted over time? We think that this might be asking too much from signalers, who seem to have trouble producing intransitive actions in imitation studies (Tennie et al., 2012). Thus, a pounding action to indicate a stone hammer might be outside of the spontaneous repertoire of primates, but if the intransitive action could be scaffolded with transitive elements, it might be possible that primates could gesture with intended meaning using novel signals.

In this potential experimental arrangement, with the possibility of scaffolded novel gestures, it is possible to examine whether signalers would take the context into account when producing their signals, which would suggest an awareness of the inferences they can reasonably expect the recipient to make. If the context already provides enough information about their intended meaning, would their signals become less specific, particularly when more specific signals are costlier to produce? Conversely, if contextual cues are ambiguous, would signals become more specific? There exists some experimental evidence that apes use pointing variations to disambiguate between two food items when the higher-value food was placed behind a lower value food and subjects were asked to select their preferred food, *via* pointing (Tauzin et al., 2020). A similar paradigm could investigate whether apes use modified pointing gestures to disambiguate between choices where the context is identical, but their knowledge of the recipients’ past experiences or preferences is varied. For example, if one experimenter is known to always provide the higher-value food regardless of the spatial arrangement of the plates, but another experimenter is new to the situation and has no expected pattern of behavior, will the subject use modified pointing gestures to disambiguate their choice with the new experimenter, but not the familiar one?

We now turn our attention to criteria for determining which taxa and which species might be more likely to display inferential communication. Based on the examples that we have given, primates, and particularly great apes seem suitable candidates to investigate the existence of inferential communication. While the communicative behavior of many species is sufficiently captured by explanations found in the foundational models of communication, it is possible that other species, apart from primates, may also be capable of inferential communication provided they possess the required cognitive prerequisites. We propose three such prerequisites: goal-directed communication, general inferential reasoning abilities, and non-communicative social inference. If evidence of these abilities is found in any species, regardless of taxa, it is possible that inferential communication may be within their capacity as well. For example, there is a body of evidence that canines exhibit intentional communication (e.g., Rossi and Ades, 2008) and social inference (e.g., Bräuer et al., 2006). African gray parrots have been shown to exhibit general inferential abilities (e.g., Schloegl et al., 2012; Pepperberg et al., 2013, 2018), and there is some evidence to suggest that they possess the capacity for intentional communication as well (e.g., Pepperberg, 2004). These groups may, therefore, be promising candidates for inferential communication, but rigorous testing of the above pre-requisites would be necessary before investigations of inferential communication could be practically conducted in any of these groups. We do not suggest that any species meeting these criteria is *de facto* likely to use inferential communication, we merely suggest that possession of these prerequisites may serve to determine whether that species is worth closer investigation.

In sum, we have proposed several ways by which inferential communication can be used to investigate inferred meaning by recipients using true inference – the integration of information to be applied to a new scenario – as well as contextual clues based not only on situational factors, but also on the mental state of the signaler. Furthermore, we have highlighted some tasks already present in the literature that we believe test for intended meaning on the part of a signaler, and proposed ways that they could be modified to new tasks to investigate whether primates can integrate simple theory of mind into their accounting of context, and whether signalers can account for such context while producing more complex (in terms of iconicity) forms of communication. We also indicated that a species possessing goal-directed communication, general inferential reasoning abilities, and/or non-communicative social inference (with all three abilities constituting the strongest foundation) would be a good starting point to investigate inferential communication.

## Concluding Remarks

The field of animal communication has made considerable progress since the appearance of the early ethological models purely based on behavior. Much of this progress has occurred as a consequence of the development of cognitive models of animal communication. In what has otherwise been a progressive increase in cognitive sophistication aimed at explaining flexible communication, we think that the field now runs a risk of stagnation due to the rejection of any form of mental state attribution in communication (Townsend et al., 2017). In this paper we have argued that we need a more thorough and detailed understanding of mentalizing in communication, particularly for species that are flexible communicators, and especially when those data are subsequently used to make inferences about the evolution of language. Without mentalizing in models of animal communication, the gap between animal and human communication might be too wide to bridge.

We submit the model of inferential communication as a way forward – a way to progress from descriptions of potential cognitive outcomes to considerations of the actual cognitive mechanisms driving them. Evidence of cognitive forms of communication in primates, especially intentional and referential exchanges, combined with evidence of social inference such as goal attribution, leads us to propose that primates (and perhaps other species too) may have the capacity to make inferences within communicative exchanges. The idea of inference playing a role in animal communication is not new, but we argue that its potential importance and scope has not been fully realized because inference has often been conflated with other mechanisms. Moreover, we propose that investigation of inferences involving the integration of disparate pieces of information, some not based on contextual cues, may provide new insights into the mechanisms underlying the complexity and flexibility of primate communication. Our model invites a rich interpretation of the cognitive mechanisms surrounding communication by challenging the idea that meaning is drawn exclusively from a set of rules or semantics, or from conditional discrimination between situations, which might otherwise suggest simplistic associative learning or hardwired signal-response connections. We also decouple informative intention from communicative intention and suggest that it is possible for actors in a communicative exchange to engage with simple mental state attribution and expression of goals, absent the recursive levels of theory of mind found in ostensive models of communication.

The model of inferential communication is a multi-level framework, beginning with social inferences regarding non-communicative behavior and extending to communicative inferences regarding how signals are used and interpreted, including consideration of the motivation underlying communicative exchanges. With regard to signals, we have illustrated the inferential approach to interpreting ambiguous signals, re-purposing old signals, and creating new ones. Each level shares the fundamental requirement that both individuals, the signaler and the recipient, must make leaps of interpretation for successful communication. For some of these levels, there is already some evidence suggesting that primates might be capable of communicative inferences, but for other levels there is only anecdotal or even negative evidence. Furthermore we have proposed ways in which these ideas could be tested using new tasks or by modifying existing ones. With regard to motivation we have argued that a prosocial motivation is not strictly necessary for this form of communication to arise because it can hijack other motivational systems for the same successful outcome, but if present, it may facilitate successful communication involving the production of novel and initially opaque signals.

Our proposal extends beyond current approaches to referential and intentional communication but stops short of ostensive communication. Although we do not rule out *a priori* the possibility that ostensive communication could occur in primates, we suggest that before tackling this issue, is important to explore the possibility of inferential communication, which is in some ways a pre-requisite for ostensive communication. Our proposal therefore does not qualify as mentalistic communication in the Gricean sense (Grice, 1957, 1969, 1989) but unlike Townsend et al. (2017) it does not flatly reject the importance of some forms of mentalizing, which we incorporate to our model. Namely, we argue that goal attribution, visual perspective taking, and knowledge attribution may play an important role in the inferences that individuals make in their communicative exchanges.

Finally, our endorsement of inferential communication should not be taken as an indication that we believe primates engage in inferential communication in every communicative exchange. Instead, we propose that individuals mainly engage inferential communication when routine conditions change, and new solutions are required. In this sense, engaging inferential communication is analogous to engaging cognitive control and monitoring mechanisms in problem solving following the perturbation of a previously stable system. We believe that inferential communication is ideally placed to bridge the gap between the intentional and the ostensive model of communication, something that it is particularly important for those wishing to make inferences regarding the evolution of language. It is a framework that we hope will contribute to more precise descriptions of phenomena we have already witnessed in primates and promote new insights into the complexity of animal communication. It is a toolkit – a perspective that we hope will empower researchers to take a more productive approach to animal communication, both in design and interpretation.

## Author Contributions

Both authors contributed jointly to the conceptual and theoretical development of the model of communication presented in the manuscript, contributed to the writing and reviewing each draft of the manuscript, and approved the submitted version.

## Conflict of Interest

The authors declare that the research was conducted in the absence of any commercial or financial relationships that could be construed as a potential conflict of interest.

## Publisher’s Note

All claims expressed in this article are solely those of the authors and do not necessarily represent those of their affiliated organizations, or those of the publisher, the editors and the reviewers. Any product that may be evaluated in this article, or claim that may be made by its manufacturer, is not guaranteed or endorsed by the publisher.

## References

[B1] Arbib M. A. Liebal K. Pika S. (2008). Primate vocalization, gesture, and the evolution of human language. *Curr. Anthropol.* 49 1053–1076. 10.1086/593015 19391445

[B2] Bohn M. Call J. Tomasello M. (2016a). Comprehension of iconic gestures by chimpanzees and human children. *J. Exp. Child Psychol.* 142 1–17. 10.1016/j.jecp.2015.09.001 26448391

[B3] Bohn M. Call J. Tomasello M. (2016b). The role of past interactions in great apes’ communication about absent entities. *J. Comp. Psychol.* 130 351–357. 10.1037/com0000042 27690504

[B4] Bräuer J. Kaminski J. Riedel J. Call J. Tomasello M. (2006). Making inferences about the location of hidden food: social dog, causal ape. *J. Comp. Psychol.* 120 38–47. 10.1037/0735-7036.120.1.38 16551163

[B5] Bretherton I. Bates E. (1979). The emergence of intentional communication. *New Dir. Child Adolesc. Dev.* 1979 81–100. 10.1017/S0305000912000359 22883708

[B6] Buttelmann D. Carpenter M. Call J. Tomasello M. (2007). Enculturated chimpanzees imitate rationally. *Dev. Sci.* 10 F31–F38. 10.1111/j.1467-7687.2007.00630.x 17552931

[B7] Buttelmann D. Carpenter M. Call J. Tomasello M. (2008). Rational tool use and tool choice in human infants and great apes. *Child Dev.* 79 609–626. 10.1111/j.1467-8624.2008.01146.x 18489416

[B8] Buttelmann D. Schütte S. Carpenter M. Call J. Tomasello M. (2012). Great apes infer others’ goals based on context. *Anim. Cogn.* 15 1037–1053. 10.1007/s10071-012-0528-4 22752816

[B9] Byrne R. W. Cartmill E. Genty E. Graham K. E. Hobaiter C. Tanner J. (2017). Great ape gestures: intentional communication with a rich set of innate signals. *Anim. Cogn.* 20 755–769. 10.1007/s10071-017-1096-4 28502063 PMC5486474

[B10] Call J. (2001). Body imitation in an enculturated orangutan (Pongo pygmaeus). *Cybern. Syst.* 32 97–119. 10.1080/019697201300001821

[B11] Call J. Hare B. Carpenter M. Tomasello M. (2004). ‘Unwilling’ versus ‘unable’: chimpanzees’ understanding of human intentional action. *Dev. Sci.* 7 488–498. 10.1111/j.1467-7687.2004.00368.x 15484596

[B12] Call J. Tomasello M. (eds) (2007). *The Gestural Communication of Apes and Monkeys.* Oxfordshire: Taylor & Francis Group.

[B13] Call J. Tomasello M. (1998). Distinguishing intentional from accidental actions in orangutans (*Pongo pygmaeus*), chimpanzees (*Pan troglodytes*) and human children (*Homo sapiens*). *J. Comp. Psychol.* 112 192–206. 10.1037/0735-7036.112.2.192 9642787

[B14] Cartmill E. A. Hobaiter C. (2019). Developmental perspectives on primate gesture: 100 years in the making. *Anim. Cogn.* 22 453–459. 10.1007/s10071-019-01279-w 31278622

[B15] Cheney D. L. Seyfarth R. M. (2008). *Baboon Metaphysics: The Evolution of a Social Mind.* Chicago: University of Chicago Press.

[B16] Cheney D. L. Seyfarth R. M. (2018). Flexible usage and social function in primate vocalizations. *Proc. Natl. Acad. Sci. U. S. A.* 115 1974–1979. 10.1073/pnas.1717572115 29432157 PMC5834704

[B17] Cronin K. A. (2012). “Cognitive aspects of prosocial behavior in nonhuman primates,” in *Encyclopedia of the Sciences of Learning*, ed. Seel N. M. (Boston: Springer), 581–583.

[B18] Cronin K. A. Schroeder K. K. Snowdon C. T. (2010). Prosocial behaviour emerges independent of reciprocity in cottontop tamarins. *Proc. R. Soc. B Biol. Sci.* 277 3845–3851. 10.1098/rspb.2010.0879 20630886 PMC2992700

[B19] Dawkins R. Krebs J. R. (1978). “Animal signals: information or manipulation,” in *Behavioural Ecology: An Evolutionary Approach*, eds Krebs J. R. Davies N. B. (Oxford: Blackwell), 282–309.

[B20] Eckert J. Rakoczy H. Call J. Herrmann E. Hanus D. (2018). Chimpanzees consider humans’ psychological states when drawing statistical inferences. *Curr. Biol.* 28 1959–1963. 10.1016/j.cub.2018.04.077 29861138

[B21] Fischer J. (2013). “Information, inference and meaning in primate vocal behaviour,” in *Animal Communication Theory: Information and Influence*, ed. Stegmann U. (Cambridge: Cambridge University Press), 297–319.

[B22] Fischer J. Price T. (2017). Meaning, intention, and inference in primate vocal communication. *Neurosci. Biobehav. Rev.* 82 22–31. 10.1016/j.neubiorev.2016.10.014 27773691

[B23] Fitch W. T. (2015). Evolving pragmatics. *Curr. Biol.* 25 R1110–R1112.

[B24] Fouts R. S. (1972). Use of guidance in teaching sign language to a chimpanzee (*Pan troglodytes*). *J. Comp. Physiol. Psychol.* 80 515–522. 10.1037/h0032989

[B25] Gardner R. A. Gardner B. T. Van Cantfort T. E. (eds) (1989). *Teaching Sign Language to Chimpanzees.* New York: Suny Press.

[B26] Gómez J. C. (1990). “The emergence of intentional communication as a problem-solving strategy in the gorilla,” in *Language” and Intelligence in Monkeys and Apes*, eds Parker S. T. Gibson K. R. (Cambridge: Cambridge University Press), 333–355.

[B27] Gómez J. C. (2005). “Joint Attention and the Notion of Subject: Insights from Apes, Normal Children, and Children with Autism,” in *Joint Attention: Communication and Other Minds: Issues in Philosophy and Psychology*, eds Eilan N. Hoerl C. McCormack T. Roessler J. (Oxford: Oxford University Press), 65–84. 10.1093/acprof:oso/9780199245635.003.0004

[B28] Graham K. E. Wilke C. Lahiff N. J. Slocombe K. E. (2020). Scratching beneath the surface: intentionality in great ape signal production. *Philos. Trans. R. Soc. B* 375:20180403. 10.1098/rstb.2018.0403 31735155 PMC6895546

[B29] Grice H. P. (1957). Meaning. *Philos. Rev.* 66 377–388.

[B30] Grice H. P. (1969). Utterer’s meaning and intentions. *Philos. Rev.* 78 147–177.

[B31] Grice H. P. (1989). *Studies in the Way of Words.* Cambridge: Harvard University Press.

[B32] Grosse K. Call J. Carpenter M. Tomasello M. (2015). Differences in the ability of apes and children to instruct others using gestures. *Lang. Learn. Dev.* 11 310–330. 10.1080/15475441.2014.955246

[B33] Hare B. (2017). Survival of the friendliest: *Homo sapiens* evolved *via* selection for prosociality. *Annu. Rev. Psychol.* 68 155–186. 10.1146/annurev-psych-010416-044201 27732802

[B34] Heintz C. Scott-Phillips T. (2022). Expression Unleashed: the evolutionary & cognitive foundations of human communication. *Behav. Brain Sci.* 1–45. 10.1017/S0140525X22000012 34983701

[B35] Hewes G. W. Andrew R. J. Carini L. Choe H. Gardner R. A. Kortlandt A. (1973). Primate communication and the gestural origin of language [and comments and reply]. *Curr. Anthropol.* 14 5–24.

[B36] Hill A. Collier-Baker E. Suddendorf T. (2011). Inferential reasoning by exclusion in great apes, lesser apes, and spider monkeys. *J. Comp. Psychol.* 125 91–103. 10.1037/a0020867 21341913

[B37] Hobaiter C. Byrne R. W. (2014). The meanings of chimpanzee gestures. *Curr. Biol.* 24 1596–1600. 10.1016/j.cub.2014.05.066 24998524

[B38] Hopkins W. D. Taglialatela J. P. Leavens D. A. (2007). Chimpanzees differentially produce novel vocalizations to capture the attention of a human. *Anim. Behav.* 73 281–286. 10.1016/j.anbehav.2006.08.004 17389908 PMC1832264

[B39] Krebs J. R. Dawkins R. (1984). “Animal Signals: Mind Reading and Manipulation,” in *Behavioural Ecology: An Evolutionary Approach*, eds Krebs J. R. Davies N. B. (Oxford: Blackwell).

[B40] Leavens D. A. Hostetter A. B. Wesley M. J. Hopkins W. D. (2004). Tactical use of unimodal and bimodal communication by chimpanzees, *Pan troglodytes*. *Anim. Behav.* 67 467–476. 10.1016/j.anbehav.2003.04.007

[B41] Leavens D. A. Russell J. L. Hopkins W. D. (2005). Intentionality as measured in the persistence and elaboration of communication by chimpanzees (*Pan troglodytes*). *Child Dev.* 76 291–306. 10.1111/j.1467-8624.2005.00845.x 15693773 PMC2043155

[B42] Liebal K. Pika S. Tomasello M. (2006). Gestural communication of orangutans (*Pongo pygmaeus*). *Gesture* 6 1–38.

[B43] Liebal K. Waller B. M. Slocombe K. E. Burrows A. M. (2014). *Primate Communication: A Multimodal Approach.* Cambridge: Cambridge University Press.

[B44] Lorenz K. Z. (1966). Evolution of ritualization in the biological and cultural spheres. *Philos. Trans. R. Soc. Lond. B Biol. Sci.* 251 273–284. 10.1017/S0007087415000035 26256313

[B45] Marler P. (1961). The logical analysis of animal communication. *J. Theor. Biol.* 1:295e317. 10.1016/0022-5193(61)90032-7 13766984

[B46] Massen J. J. Van Den Berg L. M. Spruijt B. M. Sterck E. H. (2010). Generous leaders and selfish underdogs: pro-sociality in despotic macaques. *PLoS One* 5:e9734. 10.1371/journal.pone.0009734 PMC2840023 20305812

[B47] Moore R. (2016). Meaning and ostension in great ape gestural communication. *Anim. Cogn.* 19 223–231. 10.1007/s10071-015-0905-x 26223212

[B48] Moore R. (2017). Convergent minds: ostension, inference and Grice’s third clause. *Interface Focus* 7:20160107. 10.1098/rsfs.2016.0107 28479978 PMC5413889

[B49] Owren M. J. Rendall D. Ryan M. J. (2010). Redefining animal signaling: influence versus information in communication. *Biol. Philos.* 25 755–780. 10.1007/s10539-010-9224-4

[B50] Pelé M. Dufour V. Thierry B. Call J. (2009). Token transfers among great apes (*Gorilla gorilla*, *Pongo pygmaeus*, *Pan paniscus*, and *Pan troglodytes*): species differences, gestural requests, and reciprocal exchange. *J. Comp. Psychol.* 123 375–84. 10.1037/a0017253 19929106

[B51] Pepperberg I. M. (2004). “Insightful” string-pulling in Grey parrots (*Psittacus erithacus*) is affected by vocal competence. *Anim. Cogn.* 7 263–266. 10.1007/s10071-004-0218-y 15045620

[B52] Pepperberg I. M. Gray S. L. Mody S. Cornero F. M. Carey S. (2018). Logical reasoning by a Grey parrot? A case study of the disjunctive syllogism. *Behaviour* 156 409–445. brill.com/beh 10.1163/1568539X-00003528

[B53] Pepperberg I. M. Koepke A. Livingston P. Girard M. Hartsfield L. A. (2013). Reasoning by inference: further studies on exclusion in grey parrots (*Psittacus erithacus*). *J. Comp. Psychol.* 127 272–281. 10.1037/a0031641 23421751

[B54] Petit O. Dufour V. Herrenschmidt M. De Marco A. Sterck E. H. Call J. (2015). Inferences about food location in three cercopithecine species: an insight into the socioecological cognition of primates. *Anim. Cogn.* 18 821–830. 10.1007/s10071-015-0848-2 25697970

[B55] Pika S. Liebal K. Call J. Tomasello M. (2005). Gestural communication of apes. *Gesture* 5 41–56. 10.4324/9781003064541-3

[B56] Plooij F. X. Lock A. (1978). “Some Basic Traits of Language in Wild Chimpanzees?,” in *Action, gesture, and symbol: The emergence of language*, ed. Lock A. (London: Academic Press).

[B57] Premack D. Premack A. J. (1994). Levels of causal understanding in chimpanzees and children. *Cognition* 50 347–362. 10.1016/0010-0277(94)90035-3 8039368

[B58] Rossi A. P. Ades C. (2008). A dog at the keyboard: using arbitrary signs to communicate requests. *Anim. Cogn.* 11 329–338. 10.1007/s10071-007-0122-3 18000692

[B59] Savage-Rumbaugh E. S. McDonald K. Sevcik R. A. Hopkins W. D. Rubert E. (1986). Spontaneous symbol acquisition and communicative use by pygmy chimpanzees (*Pan paniscus*). *J. Exp. Psychol.* 115 211–235. 10.1037//0096-3445.115.3.211 2428917

[B60] Schel A. M. Machanda Z. Townsend S. W. Zuberbühler K. Slocombe K. E. (2013). Chimpanzee food calls are directed at specific individuals. *Anim. Behav.* 86 955–965. 10.1371/journal.pone.0144197 26909518 PMC4766192

[B61] Schloegl C. Schmidt J. Boeckle M. Weiß B. M. Kotrschal K. (2012). Grey parrots use inferential reasoning based on acoustic cues alone. *Proc. R. Soc. B Biol. Sci.* 279 4135–4142. 10.1098/rspb.2012.1292 22874753 PMC3441070

[B62] Scott-Phillips T. (2014). *Speaking Our Minds: Why Human Communication is Different, and How Language Evolved to Make it Special.* London: Macmillan International Higher Education.

[B63] Scott-Phillips T. C. (2015). Nonhuman primate communication, pragmatics, and the origins of language. *Curr. Anthropol.* 56 56–80. 10.1086/679674

[B64] Seyfarth R. M. Cheney D. L. (2010). Production, usage, and comprehension in animal vocalizations. *Brain Lang.* 115 92–100. 10.1016/j.bandl.2009.10.003 19944456

[B65] Seyfarth R. M. Cheney D. L. (2017). Precursors to language: social cognition and pragmatic inference in primates. *Psychon. Bull. Rev.* 24 79–84. 10.3758/s13423-016-1059-9 27368618

[B66] Seyfarth R. M. Cheney D. L. Marler P. (1980). Monkey responses to three different alarm calls: evidence of predator classification and semantic communication. *Science* 210 801–803. 10.1126/science.7433999 7433999

[B67] Shannon C. E. Weaver W. (1949). *The Mathematical Theory of Communication.* Urbana: University of Illinois Press.

[B68] Shorland G. (2018). *The self and others: A glimpse into the bonobo mind.* Ph.D thesis. Switzerland: Université de Neuchâtel.

[B69] Slocombe K. E. Zuberbühler K. (2005). Functionally referential communication in a chimpanzee. *Curr. Biol.* 15 1779–1784. 10.1016/j.cub.2005.08.068 16213827

[B70] Smith W. J. (1977). *The Behavior of Communicating.* Cambridge: Harvard University Press.

[B71] Sperber D. Wilson D. (1986). *Relevance: Communication and Cognition.* Cambridge: Harvard University Press.

[B72] Tauzin T. Bohn M. Gergely G. Call J. (2020). Context-sensitive adjustment of pointing in great apes. *Sci. Rep.* 10:1048. 10.1038/s41598-019-56183-7 31974479 PMC6978377

[B73] Tennie C. Call J. Tomasello M. (2009). Ratcheting up the ratchet: on the evolution of cumulative culture. *Philos. Trans. R. Soc. B Biol. Sci.* 364 2405–2415. 10.1098/rstb.2009.0052 19620111 PMC2865079

[B74] Tennie C. Call J. Tomasello M. (2012). Untrained chimpanzees (Pan troglodytes schweinfurthii) fail to imitate novel actions. *PLoS One* 7:e41548. 10.1371/journal.pone.0041548 PMC3414512 22905102

[B75] Tinbergen N. (1952). Derived activities; their causation, biological significance, origin, and emancipation during evolution. *Q. Rev. Biol.* 27 1–32. 10.1086/398642 14930222

[B76] Tolman E. C. (1932). *Purposive Behavior in Animals and Men.* Berkeley: University of California Press.

[B77] Tomasello M. (2008). Why don’t apes point?. *Trends Linguist. Stud. Monogr.* 197:375.

[B78] Tomasello M. (2009). “Primate communication,” in *Key Notions for Pragmatics*, eds Verschueren J. Östman J.-O. (Amsterdam: John benjamins publishing company), 208–216.

[B79] Tomasello M. Call J. (2019). Thirty years of great ape gestures. *Anim. Cogn.* 22 461–469. 10.1007/s10071-018-1167-1 29468285 PMC6647417

[B80] Tomasello M. Call J. Warren J. Frost G. T. Carpenter M. Nagell K. (1997). The ontogeny of chimpanzee gestural signals: a comparison across groups and generations. *Evol. Commun.* 1 223–259. 10.1075/eoc.1.2.04tom

[B81] Tomasello M. George B. L. Kruger A. C. Jeffrey M. Evans A. (1985). The development of gestural communication in young chimpanzees. *J. Hum. Evol.* 14 175–186. 10.1016/s0047-2484(85)80005-1

[B82] Tomasello M. Gust D. Frost G. T. (1989). A longitudinal investigation of gestural communication in young chimpanzees. *Primates* 30 35–50. 10.1111/j.1540-5834.2005.00324.x 16156847

[B83] Tomasello M. Zuberbühler K. (2002). “Primate vocal and gestural communication,” in *The Cognitive Animal: Empirical and theoretical perspectives on animal cognition*, eds Bekoff M. Allen C. Burghardt G. M. (Cambridge: MIT Press), 293–299.

[B84] Townsend S. W. Koski S. E. Byrne R. W. Slocombe K. E. Bickel B. Boeckle M. (2017). Exorcising G rice’s ghost: an empirical approach to studying intentional communication in animals. *Biol. Rev.* 92 1427–1433. 10.1111/brv.12289 27480784

[B85] Vail A. L. Manica A. Bshary R. (2013). Referential gestures in fish collaborative hunting. *Nat. Commun.* 4:1765. 10.1038/ncomms2781 23612306

[B86] Völter C. J. Call J. (2017). “Causal and inferential reasoning in animals,” in *APA handbook of comparative psychology: Perception, learning, and cognition*, eds Call J. Burghardt G. M. <suffix>I</suffix>, Pepperberg M. Snowdon C. T. Zentall T. (Washington: American Psychological Association), 643–671. 10.1037/0000012-029

[B87] Wilson D. (1998). “Linguistic structure and inferential communication,” in *Proceedings of the 16th International Congress of Linguists 20-25* (Oxford: Elsevier Sciences).

[B88] Wilson D. Sperber D. (2002). “Relevance theory,” in *The Handbook of Pragmatics*, eds Horn L. Ward G. (Oxford: Blackwell), 607–632.

[B89] Woodruff G. Premack D. (1979). Intentional communication in the chimpanzee: the development of deception. *Cognition* 7 333–362.

[B90] Yamamoto S. Humle T. Tanaka M. (2012). Chimpanzees’ flexible targeted helping based on an understanding of conspecifics’ goals. *Proc. Natl. Acad. Sci. U. S. A.* 109 3588–3592. 10.1073/pnas.1108517109 22315399 PMC3295314

[B91] Zuberbühler K. (2005). The phylogenetic roots of language: evidence from primate communication and cognition. *Curr. Dir. Psychol. Sci.* 14 126–130.

